# Two new rare species of *Candolleomyces* with pale spores from China

**DOI:** 10.3897/mycokeys.80.67166

**Published:** 2021-06-03

**Authors:** Tolgor Bau, Jun-Qing Yan

**Affiliations:** 1 Key Laboratory of Edible Fungal Resources and Utilization (North), Ministry of Agriculture and Rural Affairs, Jilin Agricultural University, Changchun 130118, China Jilin Agricultural University Changchun China; 2 Jiangxi Key Laboratory for Conservation and Utilization of Fungal Resource, Jiangxi Agricultural University, Nanchang, Jiangxi 330045, China Jiangxi Agricultural University Nanchang China

**Keywords:** Basidiomycete, new taxon, Psathyrellaceae, phylogenetic analysis, taxonomy

## Abstract

Most species of *Candolleomyces* have brown or dark brown spores. Although pale-spored members are rare in the genus we frequently collected two such species from many Provinces during our investigations in subtropical China from 2016–2020. As revealed by morphological characterisation and multigene phylogenetic analyses (ITS LSU *β-tub* and *tef*-*1α*) these species which we have named *C. subcacao* and *C. subminutisporus* are unique and distinct from known taxa. In addition a new combination *C. cladii-marisci* is proposed on the basis of ITS sequence analysis of the type specimen. Detailed descriptions colour photos illustrations and a key to related species are presented.

## Introduction

On the basis of extensive comparisons of gene sequences and phylogenetic analyses, the historical genus *Psathyrella* (Fr.) Quél. has been split into several genera. One of these genera is *Candolleomyces* D. Wächt. & A. Melzer, which differs from *Psathyrella* s.s. in lacking pleurocystidia (Örstadius et al. 2015; Wächter and Melzer 2020). Approximately 100 taxa (including synonyms and subspecies) without pleurocystidia have been previously described in *Psathyrella* s.l. (Fries 1838; Smith 1972; Kits van Waveren 1985; Örstadius and Kundsen 2012; Battistin et al. 2014); however, many of these taxa have been treated as synonyms, as unique features, based on conventional methods, are scarce (Galland et al. 1979; Kits van Waveren 1980; Knudsen and Vesterholt 2012). Currently, 26 species have been assigned to *Candolleomyces* (Wächter & Melzer, 2020).

According to the research of Wächter and Melzer (2020), *Candolleomyces* may be more speciose than previously thought and better delimitation of species boundaries is needed. Although controversies still exist regarding some species boundaries, the number of new taxa is steadily increasing (Melzer et al. 2018; Sicoli et al. 2019a; Büttner et al. 2020). This continuous discovery of new taxa with clear boundaries deepens understanding of the species in this genus.

Approximately eight taxa in the genus *Candolleomyces* have previously been reported from China (Yan 2018). During our investigations in subtropical China from 2016–2020, we frequently collected two unknown *Candolleomyces* species with pale spores from many Provinces. Spores that are pale or almost colourless in water and 5% potassium hydroxide (KOH) are very rare in this genus. On the basis of our morphological and phylogenetic analyses, the specimens are described as new species in this paper.

## Materials and methods

### Morphological studies

Specimens were deposited in the Herbarium of Mycology, Jilin Agricultural University (**HMJAU**) and the Herbarium of Fungi, Jiangxi Agricultural University (**HFJAU**). Macromorphological characters and habitat details were recorded from fresh basidiomata. Colour codes were based on the Methuen Handbook of Colour (Kornerup and Wanscher 1978). More than 30 spores, cystidia and basidia in water and 5% aqueous KOH were measured under a microscope. In subsequent descriptions, measurements are shown as (*a*)*b*–*c* (*d*), where *a* is the lowest value, *b*–*c* encompasses at least 90% of values and *d* is the highest value, while *Q* is the length–width ratio of a spore (Bas 1969; Yu et al. 2020).

### DNA extraction and sequencing

DNA was extracted from dried specimens using a NuClean Plant Genomic DNA kit (CWBIO, China). Four DNA regions (ITS, LSU, *Tef-1α* and *β-tub*) were selected for analysis (Örstadius et al. 2015) and were respectively amplified using the primer pairs ITS1/ITS4 (White et al. 1990), LR0R/LR7 (Hopple and Vilgalys 1999), EF983F/EF2218R (Örstadius et al. 2015) and B36f/B12r (Nagy et al. 2011). PCR amplifications were performed using the following touchdown programme: 5 min at 95 °C, followed by 15 rounds of 1 min at 95 °C, 30 s at 65 °C (lowered by 1 °C per cycle) and 1 min at 72 °C, followed by 20 rounds of 1 min at 95 °C, 30 s at 50 °C and 1 min at 72 °C, with a final extension of 10 min at 72 °C (Yan and Bau 2018b). Sequencing was carried out by Qing Ke Biotechnology Co. (Wuhan, China).

### Data analyses

Taking into consideration the results of BLAST searching against GenBank and the research of Büttner et al. (2020) and Wächter and Melzer (2020), we analysed ITS, LSU, *tef-1α* (Tef 1^st^, Tef 2^nd^ and Tef 3^rd^) and *β-tub* (Tub 1^st^ and Tub 2^nd^) sequences from 37 taxa. Details are presented in Table 1. Sequences were aligned using the online version of the multiple sequence alignment programme MAFFT v.7 (Katoh and Standley 2013), followed by manual adjustment in BIOEDIT v.7.1.3.0 (Hall 1999). Phylogenetic analyses were conducted using Bayesian Inference (BI) in MrBayes v.3.2.6 (Ronquist et al. 2012) and by Maximum Likelihood (ML) in IQTREE v.1.5.6 (Nguyen et al. 2014). For the BI analyses, four Monte Carlo Markov chains were run for 10 million generations, with sampling every 100^th^ generation and with the first 25% of trees discarded as burn-in (Ronquist et al. 2012). ML analyses were undertaken by applying the ultrafast bootstrap approximation with 1000 replicates. The sequence alignment has been deposited in TreeBASE (S28074).

**Table 1.** Sequences used in this study.

Taxa	Voucher	Locality	ITS	LSU	*β-Tub*	*tef-1α*
*Candolleomyces albipes*	DED8340	Sao Tome	KX017209	–	–	–
*C. aberdarensis*	GLM-F116094	Kenya	MH880928	–	–	–
*C. badhyzensis*	79478 (TAA) Type	Turkmenistan	KC992883	KC992883	–	–
*C. badiophyllus*	SZMC-NL-2347	–	FN430699	FM876268	FN396261	FM897252
*C. cacao*	SFSU DED 8339	Sao Tome	NR148106	–	–	–
	FP1R4	USA	KU847452	–	–	–
	MP2R2	USA	KU847436	–	–	–
*C. candolleanus*	LAS73030 Neotype	Sweden	KM030175	KM030175	–	–
*C. efflorescens*	Pegler2133 (K)	Sri Lanka	KC992941	–	–	–
*C. eurysporus*	GLM-F126263 Type	Germany	MT651560	MT651560	–	–
*C. leucotephrus*	LÖ138-01 (UPS)	Sweden	KC992885	KC992885	KJ664865	KJ732775
*C. luteopallidus*	Sharp20863 (MICH) Type	USA	KC992884	KC992884	–	–
	HMJAU5148	China: Jilin	MG734736	**MW301084**	**MW314056**	**MW314073**
*C. secotioides*	UES2918 Type	Mexico	KR003281	KR003282	–	KR003283
*C. singeri*	HMJUA37867	China: Jilin	MG734718	**MW301088**	**MW314059**	**MW314077**
	HMJAU37877	China: Chongqing	**MW301073**	**MW301091**	**MW314062**	**MW314080**
*Candolleomyces* sp.	BAB-4773	India	KP686450	–	–	–
	BAB-5172	India	KR349656	–	–	–
	BAB-4748	India	KR154977	–	–	–
	BAB-4747	India	KR154976	–	–	–
	BAB-5202	India	KT188611	–	–	–
*C. subcacao*	HMJAU37807 Type	China: Henan	**MW301064**	**MW301092**	**MW314063**	**MW314081**
	HMJAU37808	China: Henan	**MW301065**	**MW301093**	**MW314064**	**MW314082**
	HFJAU1014	China: Jiangxi	**MW559218**	–	–	–
	HFJAU1274	China: Jiangxi	**MW559219**	–	–	–
	HFJAU1488	China:Anhui	**MW559220**	–	–	–
*C. subminutisporus*	HMJAU37801 Type	China: Hubei	**MW301066**	**MW301094**	**MW314065**	**MW314083**
	HMJAU37916	China: Henan	**MW301067**	**MW301095**	**MW314066**	**MW314084**
*C. subsingeri*	HMJAU37811 Type	China: Jilin	MG734715	**MW301097**	**MW314067**	**MW314085**
	HMJAU37913	China: Jilin	MG734725	**MW301098**	**MW314068**	**MW314086**
*C. sulcatotuberculosus*	GB:LO55-12	–	KJ138422	KJ138422	–	–
	HFJAU1515	China: Fujian	**MW375696**	–	**MW382967**	**MW382965**
	Chiarello 07-10-2013	–	KJ138423	–	–	–
*C. trinitatensis*	TL9035 (C)	Ecuador	KC992882	KC992882	KJ664863	–
	ADK4162 (BR)	Togo	KC992886	KC992886	–	–
*Psathyrella cladii-marisci*	CLUF302 Type	Italy	MK080112			
**Outgroup**						
*Psathyrella multipedata*	LÖ237-04	Sweden	KC992888	KC992888	KJ664867	KJ732777

Note: Newly-generated sequences are in bold.

## Results

**Figure 1.**
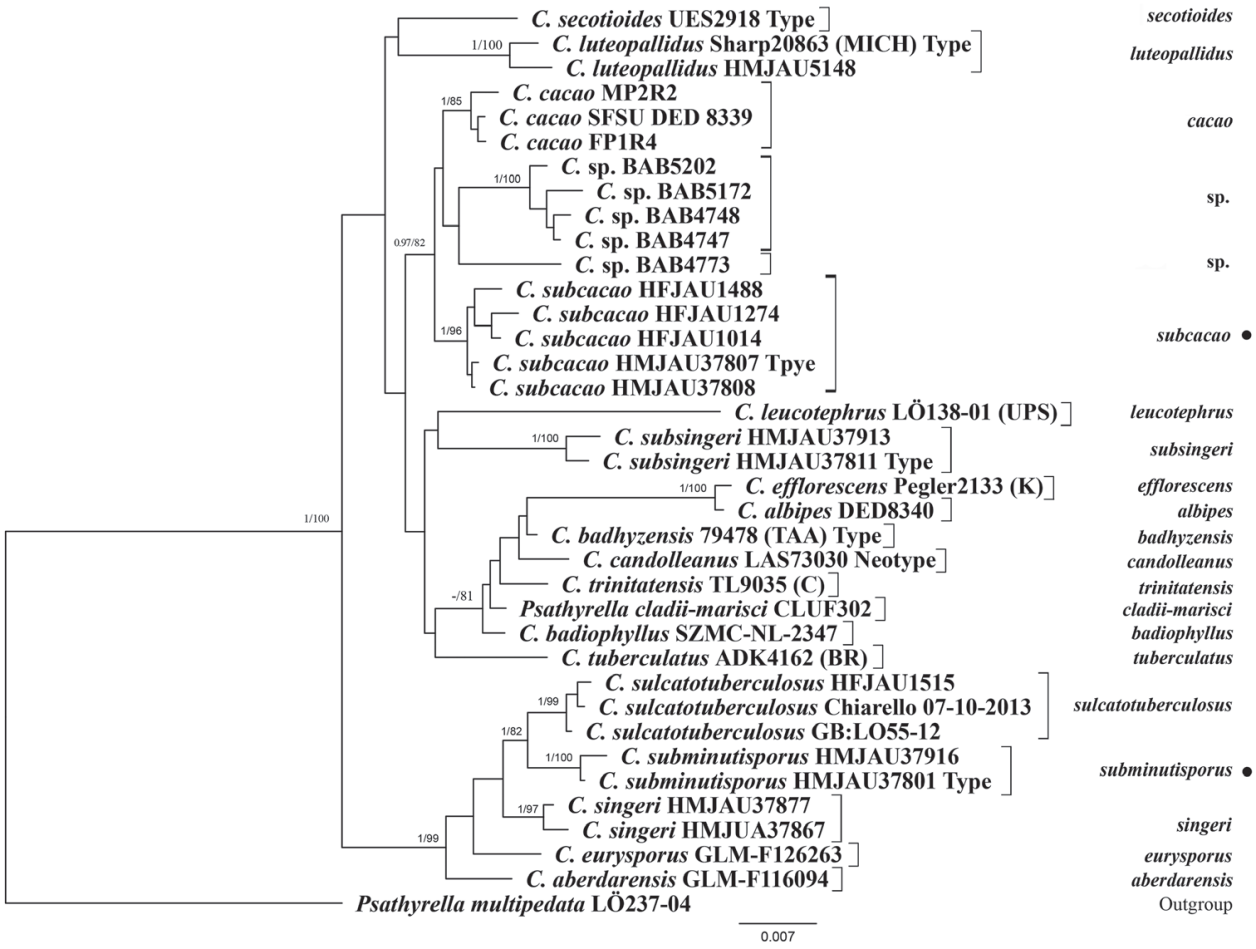
Phylogenetic tree of *Candolleomyces*. The tree was generated by Bayesian analysis of a concatenated dataset of sequences from four nuclear regions (ITS, LSU, *tef-1α* and *β-tub*). *Psathyrella multipedata* (Peck) A.H. Sm. was used as an outgroup. Bayesian posterior probabilities (BI-PP) ³ 0.95 and Maximum Likelihood bootstrap support values (ML) ≥ 75% are shown above nodes as BI-PP/ML. ● indicates newly-described species.

According to a BLAST analysis, the ITS sequence of *C. subcacao* is 98% similar (eight different loci) to that of *C. cacao* (Desjardin & B.A. Perry) D. Wächt. & A. Melzer and approximately 97% similar (19 different loci) to five ITS sequences from two unnamed species (KP686450 for BAB-4773, KR349656 for BAB-5172, KR154977 for BAB-4748, KR154976 for BAB-4747 and KT188611 for BAB-5202) isolated from *Oeceoclades maculata* (Lindley) Lindley (Bayman et al. 2016). The ITS sequence of *C. subminutisporus* shares 97% similarity (22 different loci) with that of *C. sulcatotuberculosus* (J. Favre) D. Wächt. & A. Melzer. The generated BI and ML trees are shown in Fig. 1 and Suppl. material 1, respectively. In both trees, sequences of the two new species comprise strongly supported clades that are distinct from closely-related taxa. The *C. subcacao* clade groups together with *C. cacao* and two unnamed species with high statistical support, while the *C. subminutisporus* clade clusters with *C. singeri* (A.H. Sm.) D. Wächt. & A. Melzer and *C. sulcatotuberculosus*. The type sequence of *Psathyrella cladii-marisci* Sicoli, N.G. Passal., De Giuseppe, Palermo & Pellegrino is clearly nested within *Candolleomyces*, where it groups most closely, although with only weak to moderate support, with *C. badhyzensis* (Kalamees) D. Wächt. & A. Melzer, *C. badiophyllus* (Romagn.) D. Wächt. & A. Melzer and *C. candolleanus* (Fr.) D. Wächt. & A. Melzer.

### Taxonomy

#### Candolleomyces subcacao

Taxon classificationFungiAgaricalesPsathyrellaceae

T. Bau & J.Q. Yan sp. nov.

308FE87E-E2F6-5A07-8764-3A615107335C

839231

Fig. 2

##### Holotype.

China. Henan Province: Bird Island, Nanwan Lake, Xinyang City, 32°06'43.32"N, 113°06'03.06"E, 124 m elevation, 17 July 2016, Tolgor Bau, Jun-Qing Yan, HMJAU37807 (holotype!)

##### Etymology.

Referring to its morphological similarity to *C. cacao*.

##### Diagnosis.

Differs from *C. cacao* in having a distinct spore germ pore.

##### Description.

Pileus 11–35 mm, spreading hemispherically to planar, hygrophanous, brown (7E7–7E8), striate up to halfway from the margin or indistinct, becoming slightly dirty white (7B1–7B2) upon drying. Veil pale brown (7A5–7B6), thin, fibrillose, falling off easily. Context thin and very fragile, dirty white (7B1–7B2), approximately 1.0 mm thick at the centre. Lamellae 3.0–4.0 mm wide, moderately close, adnate to slightly adnexed, pale brown (C3–C4) to dark brown (7D6–7E6), saw-toothed under 20× magnification. Stipe 40–50 mm long, approximately 2.0 mm thick, white (7A1–7B1), hollow, equal, smooth, with white fibrils (7A1–7B1) at the base. Odour and taste indistinct.

Spores 6.8–8.0(8.8) × 3.9–4.9 μm, *Q* = 1.4–1.8, ellipsoid to oblong-ellipsoid, profile slightly flattened on one side, rarely phaseoliform, inamyloid, smooth, pale yellow-brown, darkening in 5% KOH, pale brown, germ pores distinct, but small, approximately 1.0 μm wide. Basidia 17–22 × 6.1–7.3 μm, clavate, hyaline, 4-spored. Pleurocystidia absent. Cheilocystidia 22–36 × 9.8–14 μm, scattered to moderately numerous, various, utriform to fusiform, with an obtuse to broadly obtuse apex, rarely subcapitate or clavate, ovoid, thin-walled. Trama of gills irregular. Pileipellis consisting of 2–3 cells in the deep layer of the subglobose cell, 20–37 μm wide.

##### Habit and habitat.

Solitary to scattered on rotten wood in oak forest.

##### Other specimens examined.

China. Henan Province: Bird Island, Nanwan Lake, Xinyang City, 17 July 2016, Tolgor Bau and Jun-Qing Yan, HMJAU37808, HMJAU37809; Borden Forest Park, Xinyang City, 17 July 2017, Jun-Qing Yan, HMJAU37898, HMJAU37899, HMJAU37900, HMJAU37948, HMJAU44554; Jiangxi Province: Jiangxi Agricultural University, Nanchang City, 3 June 2019, Jun-Qing Yan, HFJAU0716, 9 June 2019, Jun-Qing Yan, HFJAU1274; Yun Bi Feng National Forest Park, Shangrao City, 5 July 2019, HFJAU1014.

**Figure 2.**
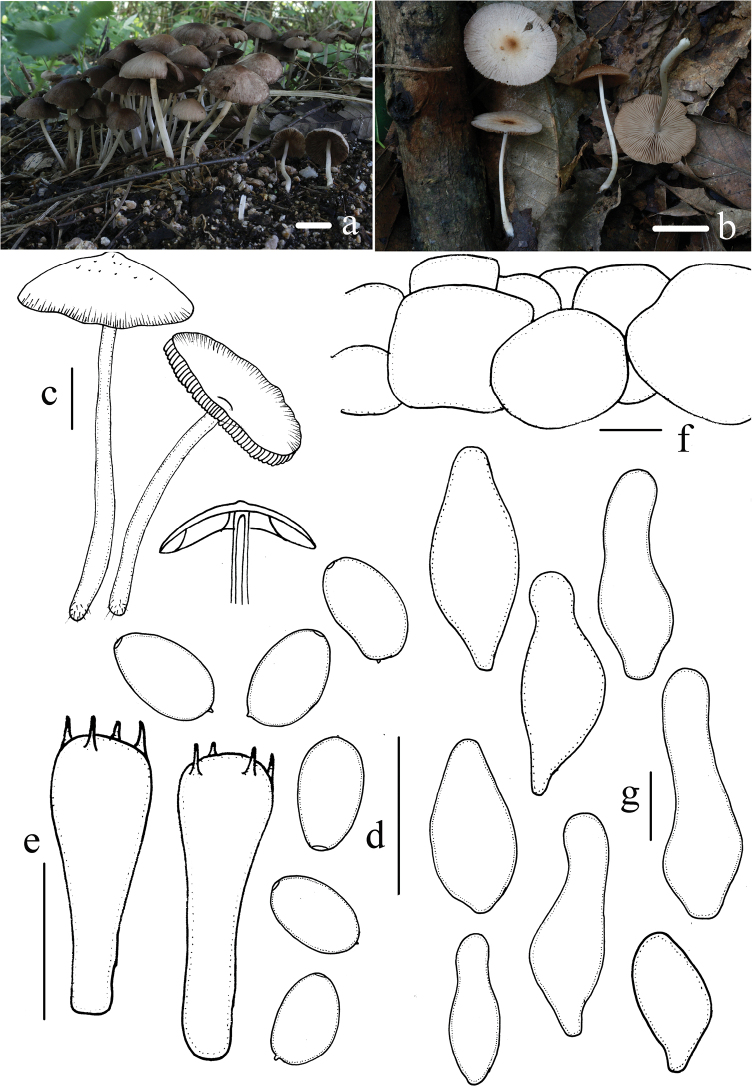
Basidiomata and microscopic features of *Candolleomyces subcacao***a–c** Basidiomata **d** spores **e** basidia **f** pileipellis **g** cheilocystidia. Scale bars: 10 mm (**a–c**); 10 μm (**d–g**).

#### Candolleomyces subminutisporus

Taxon classificationFungiAgaricalesPsathyrellaceae

T. Bau & J.Q. Yan sp. nov.

AB520731-8C1A-51A7-8D5E-1036296C5215

839232

Fig. 3

##### Etymology.

Referring to the small spores.

##### Holotype.

China. Henan Province: Boerdeng National Forest Park, Xinyang City, 16 July 2017, Tolgor Bau and Jun-Qing Yan, HMJAU37801 (holotype!).

##### Diagnosis.

Differs from *C. sulcatotuberculosus* in having smaller spores (5.8–6.8 μm long).

##### Description.

Pileus 8.0–22 mm, spreading hemispherically to broadly conical convex, hygrophanous, pale yellow-brown (6C7–6C8) at the centre, pale at the margin (6A2–6A4), striate from margin to centre, becoming pale brown (6B6–6B7) when dry. Veil present in early stages, thin, white (6A1), fibrillose, evanescent. Context thin and very fragile, 1.0–1.5 mm thick at the centre, same colour as the pileus. Lamellae 2.5–3.0 mm wide, adnate, moderately close, white (6B1) to pale coffee (6B2–6B3), edges saw-toothed under 20× magnification. Stipes 15–40 mm long, 1.0–2.0 mm thick, cylindrical, hollow, white (6B1), sometimes subhyaline or slightly yellow-brown (6A2–6B2) at the base, apex pruinose, evanescent, slightly expanded at the base. Odour and taste indistinct.

Spores 5.8–6.8(7.8) × 3.8–4.9 μm, *Q* = 1.4–1.8, ovoid, ellipsoid to oblong-ellipsoid, in profile flattened on one side, rarely phaseoliform, inamyloid, smooth, very pale, nearly hyaline in water and 5% KOH, germ pore absent. Basidia 14–20 × 7.3–7.8 μm, 4-spored, clavate, hyaline. Pleurocystidia absent. Cheilocystidia 20–32 × 11–17 μm, utriform, with obtuse apex, bottom side tapering to the long or short stipe. Caulocystidia 27–42 × 6.1–9.8 μm, present at the apex, mostly solitary, various, similar to cheilocystidia or clavate and subcapitate or not. Trama of gills irregular. Pileipellis consists of 1–2 cells in a deep layer of the subglobose cell, up to 36 μm broad.

**Figure 3.**
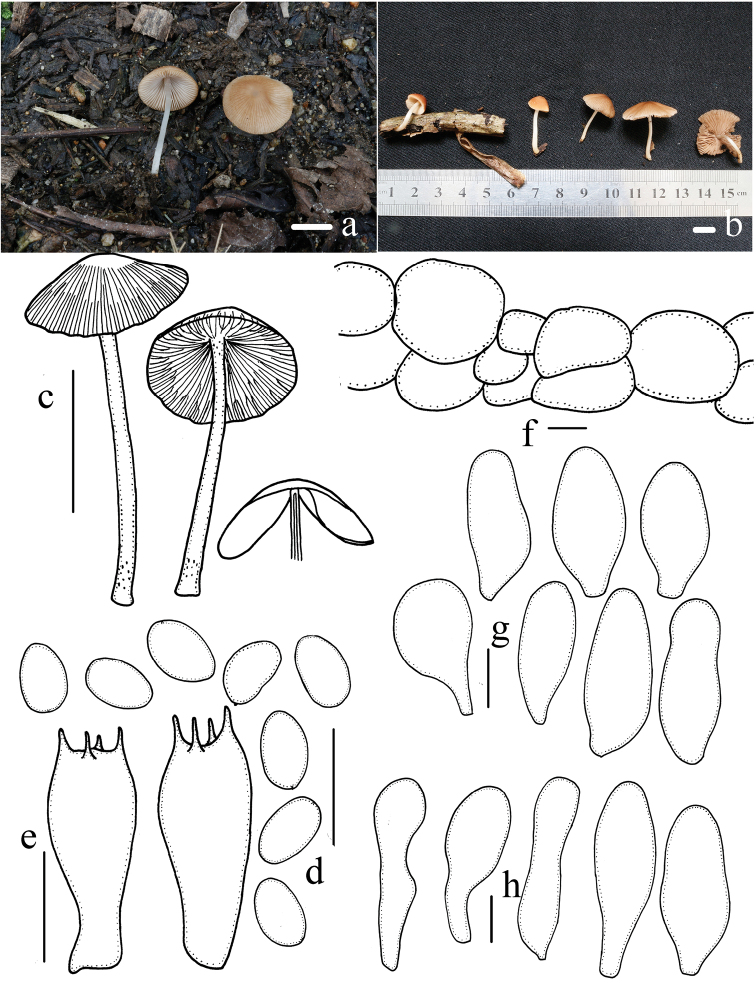
Basidiomata and microscopic features of *Candolleomyces subminutisporus***a–c** Basidiomata **d** spores **e** basidia **f** pileipellis **g** cheilocystidia **h** caulocystidia. Scale bars: 10 mm (**a–c**); 10 μm (**d–h**).

##### Habit and habitat.

Scattered on rotten wood or humus in *Pinus massoniana* and oak forests.

##### Other specimens examined.

China. Anhui Province: Huangshan City, 3 July 2018, Jun-Qing Yan, HFJAU1253, HFJAU1361; Guangxi Zhuang Autonomous Region: Qingxiushan National Forest Park, Nanning City, 12 Aug 2016, HMJAU37930; Phoenix Valley Forest Park, Nanning City, 17 Aug 2016, Jun-Qing Yan, HMJAU37950; Henan Province: Boerdeng National Forest Park, Xinyang City, 16 July 2017, Jun-Qing Yan, HMJAU37916, HMJAU37958; 17 July 2017, Jun-Qing Yan, HMJAU37959, HMJAU37960, HMJAU37961: Hubei Province: Dagui Temple National Forest Park, Suizhou City, 16 July 2016, Tolgor Bau and Jun-Qing Yan, HMJAU37800; Jiangxi Province: Lushan Mountain, Jiujiang City, 30 June 2020, Jun-Qing Yan, HFJAU0921; Yunnan Province: Kunming Botanical Garden, Kunming City, 6 Aug 2016, Jun-Qing Yan, HMJAU37929.

###### New combination

#### Candolleomyces cladii-marisci

Taxon classificationFungiAgaricalesPsathyrellaceae

(G. Sicoli, N.G. Passalacqua, A.B. De Giuseppe, A.M. Palermo & G. Pellegrino) J.Q. Yan comb. nov.

A75074AE-E5AE-5E5A-9130-60CC01034AA0

839233

Psathyrella cladii-marisci Sicoli, N.G. Passal., De Giuseppe, Palermo & Pellegrino, MycoKeys 52: 99, 2019. Basionym.

##### Note.

According to the ITS phylogenetic analysis including the type specimen, *P. cladii-marisci* belongs to *Candolleomyces* and has a close phylogenetic relationship with *C. candolleanus*, *C. badiophyllus* and *C. trinitatensis*. In addition, the morphological characteristics of this species correspond to *Candolleomyces*, which lack pleurocystidia.

For detailed descriptions and line drawings of this species, see Sicoli et al. (2019a; b).

## Discussion

Most species of *Candolleomyces* have dark brown or brown spores, whereas species with pale spores are rare. *Candolleomyces subcacao* is very easily confused with *C. cacao* in the field because of their similar macroscopic characteristics. In addition, these two species have highly similar ITS regions (98%). Nevertheless, some members of *Candolleomyces* with high ITS similarity are still treated as separate species on the basis of morphological characters (Sicoli et al. 2019a; Büttner et al. 2020; Wächter and Melzer 2020). *Candolleomyces subcacao* and *C. cacao* group together, but comprise independent lineages, in the phylogenetic tree (Fig. 1). Moreover, *C. cacao* has ventricose to broad lageniform cheilocystidia, an indistinct germ pore in 5% KOH and a tropical distribution (Desjardin 2016).

On the basis of morphology, *C. subcacao* has been classified into Psathyrella sect. Spintrigerae using the classification system of Kits van Waveren (1985; 1987) and Psathyrella sect. Subatratae, based on the system of Smith (1972). Some species in these sections lack pleurocystidia and may thus actually belong to *Candolleomyces*, but molecular analyses of type materials are needed prior to their possible reassignment. In this paper, we have, therefore, only compared these species and the new ones with respect to morphology (see the key below). In particular, two species in these sections possess the combined characteristics of small basidiomata, a pale brown and evanescent veil and pale yellow-brown spores with a distinct germ pore: *P. lacuum* Huijsman, which can be distinguished from *C. subcacao* by the presence of a veil with dispersed white arachnoid fibrils or flocci, abundant pyriform cells at the marginal of the lamellae and very rare utriform cheilocystidia (Kits van Waveren 1985; Battistin et al. 2014) and *P. cordobaensis* A.H. Sm., which differs mainly in having a 10 mm wide pileus, an indistinct germ pore and saccate to ellipsoid cheilocystidia (Smith 1972; Desjardin 2016).

*Candolleomyces subminutisporus* is characterised by the presence of small basidiomata, a pileus that is striate from the margin up to the centre and very pale to nearly hyaline spores that are mainly less than 7.0 μm long. *Candolleomyces sulcatotuberculosus* and *C. subminutisporus* are morphologically very similar and are phylogenetically closely related (Fig. 1); however, the former has a sulcate-tuberculose pileus surface and much larger spores, which measure (7.6)7.9–8.5(9) × (4.5)4.6–5.0(5.2) μm (Einhellinger 1976). Although Kits van Waveren (1985) and Battistini (2014) detected some smaller spores in these species, which measured (6.2)6.9–7.8(8.9) × (3.6)4.1–4.7(5.0) μm, most spores of *C. sulcatotuberculosus* are clearly longer than 7.0 μm.

*Candolleomyces singeri* (A.H. Sm.) D. Wächt. & A. Melzer, *C. eurysporus* A. Karich, E. Büttner & R. Ullrich and *C. aberdarensis* (A. Melzer, Kimani & R. Ullrich) D. Wächt. & A. Melzer group together with *C. subminutisporus* in the phylogenetic tree (Fig. 1). These species can be separated as follows: *C. singeri* has larger spores, mostly 6.8–7.8 μm long (Smith 1972, pers. obs. of HMJUA37867 by JQ Yan), whereas *C. eurysporus* can be separated on the basis of its broader spores, a *Q*-value of 1.2–1.6(–1.7) and brown lamellae at maturity (Büttner et al. 2020) and *C. aberdarensis* is distinguished by having larger spores [7.5–8(–8.8) μm long] (Melzer et al. 2018). In addition, two species are morphologically similar to *C. subminutisporus* in having more-or-less pale spores, germ pores that are indistinct or lacking and no pleurocystidia. These species can be separated from *C. subminutisporus* as follows: *C. halophilus* (Esteve-Rav. & Enderle) D. Wächt. & A. Melzer has larger spores, which are 8.6–11 × 4.8–6.2 μm (Esteve-Raventós and Enderle 1992; Battistin et al. 2014) and *C. subsingeri* (T. Bau & J.Q. Yan) D. Wächt. & A. Melzer is easily distinguished on the basis of its stout basidiomata (Yan and Bau 2018a).

Finally, *P. cladii-marisci* was described by Sicoli et. al. (2019) and is characterised by the absence of pleurocystidia and the presence of large spores up to 11 μm long (Sicoli et al. 2019a; b). According to our phylogenetic analysis, this species is relatively closely related to *C. candolleanus*, *C. badiophyllus* and *C. trinitatensis* and should be moved to *Candolleomyces*. A new combination is thus proposed.

### Key to related species

**Table:** 

1	Spores very pale, nearly hyaline in 5% KOH	**2**
–	Spores pale yellow-brown, greyish-brown or darker	**7**
2	Spores mostly less than 7.0 μm	**3**
–	Spores up to 8.0 μm	**4**
3	Spores broader, *Q* = 1.2–1.6, lamellae brown at maturity	***C. eurysporus***
–	Spores slenderer, *Q* = 1.4–1.8, lamellae pale coffee at maturity	***C. subminutisporus***
4	Surface of pileus is sulcate-tuberculose, up to two-thirds of the radius	***C. sulcatotuberculosus***
–	Not as above	**5**
5	Pileus less than 10 mm wide, lamellae brown	***C. aberdarensis***
–	Not as above	**6**
6	Basidiomata stout, spores up to 5.5 μm broad	**C. singeri**
–	Basidiomata slender, spores up to 4.5 μm broad	***C. subsingeri***
7	Spores up to 11 μm, growing on plant debris in brackish water	***C. halophilus***
–	Not as above	**8**
8	Germ pore distinct	**9**
–	Germ pore indistinct	**10**
9	Margin of lamellae with abundant pyriform cells, utriform cheilocystidia very rare	***P. lacuum***
–	Not as above	***C. subcacao***
10	Cheilocystidia ventricose to broadly lageniform	***C. cacao***
–	Cheilocystidia saccate to ellipsoid	***P. cordobaensis***

## Supplementary Material

XML Treatment for Candolleomyces subcacao

XML Treatment for Candolleomyces subminutisporus

XML Treatment for Candolleomyces cladii-marisci

## Supplementary materials

Supplementary material 1 Phylogram generated by Maximum Likelihood (ML) analysis Data type phylogenetic tree Explanation note Phylogram generated by Maximum Likelihood (ML) analysis of *Candolleomyces* based on sequences of a concatenated data set from four nuclear regions (ITS, LSU, *Tef-1α* and *β-tub*), rooted with *Psathyrella multipedata* (Peck) A.H. Sm. (/multipedata clade). ML bootstrap proportion (ML-BP)≥ 75% are shown. ● indicates newly described species. https://binary.pensoft.net/file/551413This dataset is made available under the Open Database License (http://opendatacommons.org/licenses/odbl/1.0/). The Open Database License (ODbL) is a license agreement intended to allow users to freely share, modify, and use this Dataset while maintaining this same freedom for others, provided that the original source and author(s) are credited.Tolgor Bau, Jun-Qing Yan

## References

[B1] Bas C (1969) Morphology and subdivision of *Amanita* and a monograph of its section Lepidella. Persoonia 5: 96–97.

[B2] Battistin E Chiarello O Vizzini A Orstadius L Larsson E (2014) Morphological characterisation and phylogenetic placement of the very rare species *Psathyrella sulcatotuberculosa*. Sydowia 66: 171–181.

[B3] Bayman P Mosquera-Espinosa AT Saladini-Aponte CM Hurtado-Guevara NC Viera-Ruiz NL (2016) Age-dependent mycorrhizal specificity in an invasive orchid, *Oeceoclades maculata*. American Journal of Botany 103: 1880–1889. 10.3732/ajb.1600127 27797713

[B4] Büttner E Karich A Nghi DH Lange M Liers C Kellner H Hofrichter M Ullrich R (2020) *Candolleomyces eurysporus*, a new Psathyrellaceae (Agaricales) species from the tropical Cúc Phương National Park, Vietnam. Austrian Journal of Mycology 28: 79–92. 10.21203/rs.3.rs-57408/v1

[B5] Desjardin DE Perry BA (2016) Dark-spored species of Agaricineae from Republic of São Tomé and Príncipe, West Africa. Mycosphere 7: 359–391. 10.5943/mycosphere/7/3/8

[B6] Einhellinger A (1976) Die Pilze in primären und sekundären Pflanzengesellschaften oberbayerischer Moore. Selbst-verlag.

[B7] Esteve-Raventós F Enderle M (1992) *Psathyrella halophila* spec. nov., eine neue Art aus der Sektion Spintrigerae (Fr.) Konrad & Maublanc vom Meeresstrand der Insel Mallorca (Spanien). Zeitschrift für Mykologie 58: 205–210.

[B8] Fries E (1838) Epicrisis Systematis Mycologici. seu synopsis Hymenomycetum. Uppsala.

[B9] Galland MC Rfo K Jurand MK (1979) The species problem in the *Psathyrella candolleana* complex. Mycotaxon 8: 329–332.

[B10] Hall TA (1999) BioEdit: a user-friendly biological sequence alignment editor and analysis program for Windows 95/98/NT. Nucleic Acids Symposium Series 41: 95–98.

[B11] Hopple JJ Vilgalys R (1999) Phylogenetic relationships in the mushroom genus *Coprinus* and dark-spored allies based on sequence data from the nuclear gene coding for the large ribosomal subunit RNA: divergent domains, outgroups, and monophyly. Molecular Phylogenetics & Evolution 13: 1–1. 10.1006/mpev.1999.0634 10508535

[B12] Katoh K Standley DM (2013) MAFFT multiple sequence alignment software version 7: improvements in performance and usability. Molecular Biology & Evolution 30: e772. 10.1093/molbev/mst010 PMC3603318 23329690

[B13] Kits van Waveren E (1980) Checklist of synonyms, varieties and forms of *Psathyrella candolleana*. Transactions of the British Mycological Society 75: 429–437. 10.1016/S0007-1536(80)80123-9

[B14] Kits van Waveren E (1985) The Dutch, French and British species of *Psathyrella*. Persoonia 2: 1–284.

[B15] Kits van Waveren E (1987) Additions to our monograph on *Psathyrella*. Thirteen new species, some revised keys, comments on other recently described species, and corrections and additions to our monograph. Persoonia-Molecular Phylogeny and Evolution of Fungi 13: 327–368.

[B16] Knudsen H Vesterholt J (2012) Funga Nordica. Agaricoid, boletoid, cyphelloid and gasteroid genera. Nordsvamp, Copenhagen.

[B17] Kornerup A Wanscher JHK (1978) The Methuen Handbook of Colour (3^rd^ ed.). Eyre Methuen Ltd. Reprint., London, 252 pp.

[B18] Melzer A Kimani VW Ullrich R (2018) *Psathyrella aberdarensis*, a new species of Psathyrella (Agaricales) from a Kenyan National Park. Austrian Journal of Mycology 27: 23–30.

[B19] Nagy LG Walther G Házi J Vágvölgyi C Papp T (2011) Understanding the Evolutionary Processes of Fungal Fruiting Bodies: Correlated Evolution and Divergence Times in the Psathyrellaceae. Systematic Biology 60: 303–317. 10.1093/sysbio/syr005 21368323

[B20] Nguyen LT Schmidt HA von Haeseler A Minh BQ (2014) IQ-TREE: a fast and effective stochastic algorithm for estimating maximum-likelihood phylogenies. Molecular Biology and Evolution 32: 268–274. 10.1093/molbev/msu300 25371430 PMC4271533

[B21] Örstadius L Kundsen H (2012) *Psathyrella* (Fr.) Quél. In: Knudsen H Vesterholt J (Eds) Funga Nordica Agaricoid, boletoid, cyphelloid and gasteroid genera.Nordsvamp, Copenhagen, 586–623.

[B22] Örstadius L Ryberg M Larsson E (2015) Molecular phylogenetics and taxonomy in Psathyrellaceae (Agaricales) with focus on psathyrelloid species: introduction of three new genera and 18 new species. Mycological Progress 14: 1–42. 10.1007/s11557-015-1047-x

[B23] Ronquist F Teslenko M Van Der Mark P Ayres DL Darling A Höhna S Larget B Liu L Suchard MA Huelsenbeck JP (2012) MrBayes 3.2: efficient Bayesian phylogenetic inference and model choice across a large model space. Systematic Biology 61: 539–542. 10.1093/sysbio/sys029 22357727 PMC3329765

[B24] Sicoli G Passalacqua NG De Giuseppe AB Palermo AM Pellegrino G (2019a) A new species of *Psathyrella* (Psathyrellaceae, Agaricales) from Italy. MycoKeys 52: 89–102. 10.3897/mycokeys.52.31415 31148934 PMC6533211

[B25] Sicoli G Passalacqua NG De Giuseppe AB Palermo AM Pellegrino G (2019b) Corrigendum: Sicoli G, Passalacqua NG, De Giuseppe AB, Palermo AM, Pellegrino G (2019) A new species of *Psathyrella* (Psathyrellaceae, Agaricales) from Italy. MycoKeys 52: 89–102. MycoKeys 58: e129. 10.3897/mycokeys.58.38856 PMC6533211 31148934

[B26] Smith AH (1972) The North American species of *Psathyrella*. The New York Botanical Garden 24: 1–633.

[B27] Wächter D Melzer A (2020) Proposal for a subdivision of the family Psathyrellaceae based on a taxon-rich phylogenetic analysis with iterative multigene guide tree. Mycological Progress 19: 1151–1265. 10.1007/s11557-020-01606-3

[B28] White TJ Bruns TD Lee SB Taylor JW Innis MA Gelfand DH Sninsky JJ (1990) Amplification and direct sequencing of Fungal Ribosomal RNA Genes for phylogenetics. Academic Press, San Diego, 315–322. 10.1016/B978-0-12-372180-8.50042-1

[B29] Yan JQ (2018) Taxonomy and Molecular Phylogeny of *Psathyrella* and related genera in China, Jiangxi Agricultural University.

[B30] Yan JQ Bau T (2018a) The Northeast Chinese species of *Psathyrella* (Agaricales, Psathyrellaceae). MycoKeys 33: 1–85. 10.3897/mycokeys.33.24704 PMC5911684 29686502

[B31] Yan JQ Bau T (2018b) *Psathyrella alpina* sp. nov. (Psathyrellaceae, Agaricales), a new species from China. Phytotaxa 349: 85–91. 10.11646/phytotaxa.349.1.11

[B32] Yu WJ Chang C Qin LW Zeng NK Wang SX Fan YG (2020) *Pseudosperma citrinostipes* (Inocybaceae), a new species associated with Keteleeria from southwestern China. Phytotaxa 450: 8–16. 10.11646/phytotaxa.450.1.2

