# Supplementary figures and images for: Two new rare species of Candolleomyces with pale spores from China
Source: MycoKeys. 2021 Jun 3;80:149–61. doi: 10.3897/mycokeys.80.67166 (PMC8192403; doi:10.3897/mycokeys.80.67166)

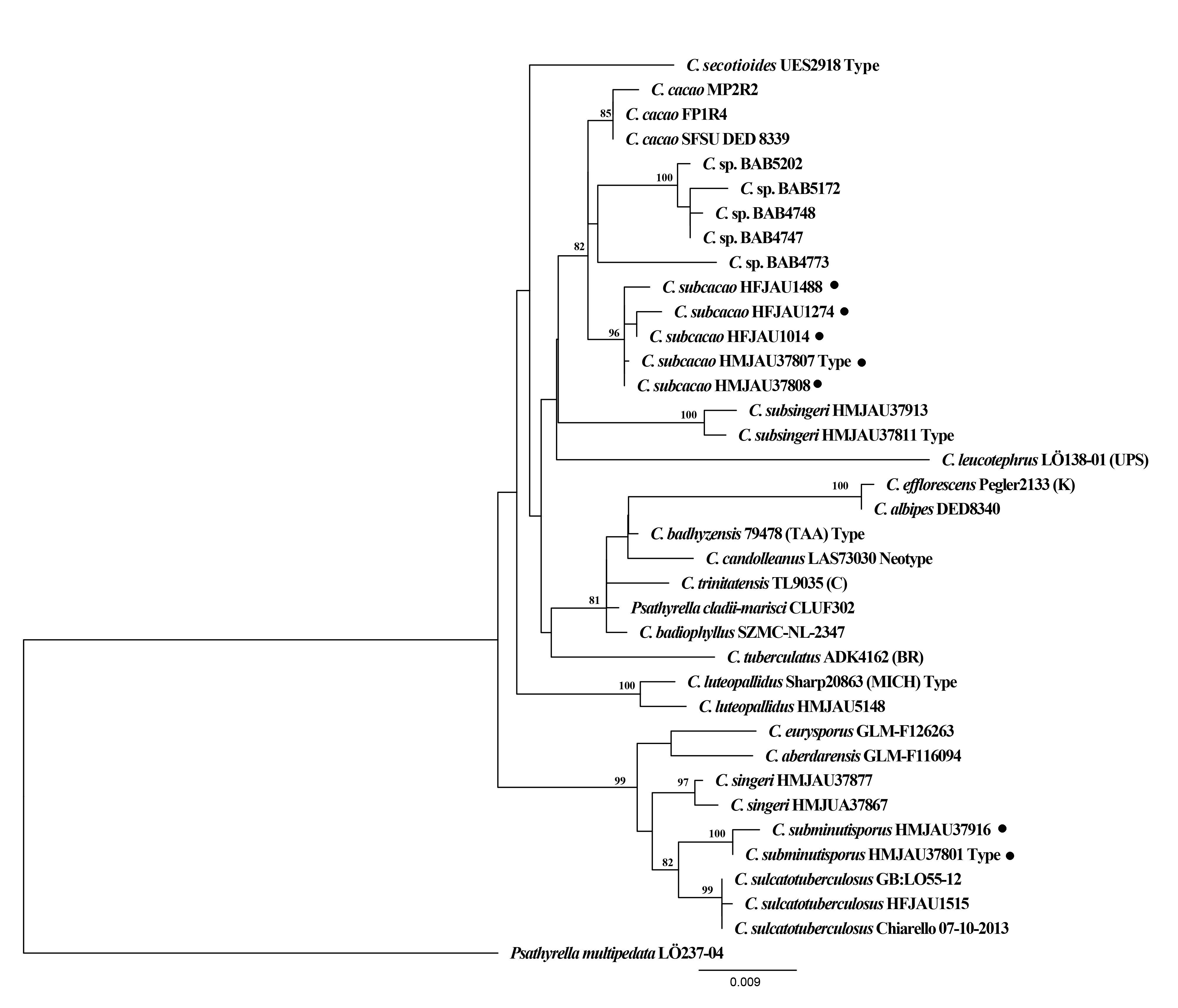

Supplement: Supplementary material 1 — Phylogram generated by Maximum Likelihood (ML) analysis [file mycokeys-80-149-s001.tif]
